# Dapagliflozin ameliorates Alzheimer’s disease in rats *via* stimulating the protective arm of renin angiotensin system and suppressing neuroinflammation

**DOI:** 10.1007/s10787-026-02345-9

**Published:** 2026-07-24

**Authors:** Zeinab M. Awwad

**Affiliations:** Department of Pharmacology and Therapeutics, Faculty of Pharmacy, Pharos University in Alexandria, Canal El Mahmoudia Street, Beside Green Plaza Complex 21648, Alexandria, Egypt

**Keywords:** Dapagliflozin, Scopolamine, Alzheimer’s disease, Renin angiotensin system, Neuroinflammation

## Abstract

Alzheimer’s disease (AD) is associated with renin angiotensin system (RAS) overactivation that stimulates amyloid β-induced neurodegeneration. Activation of angiotensin II type 1 (AT_1_) receptor impairs the protective phosphoinositide 3-kinase/protein kinase B (PI3K/Akt) pathway. The combination between angiotensin converting enzyme 2 (ACE2)/ angiotensin 1–7 (Ang 1–7) /Mas receptor (MAS1) axis activation and PI3K/Akt stimulation counteracts AT_1_-induced apoptosis. Dapagliflozin (DAPA) alters the imbalance between the two RAS arms favoring the protective arm. Accordingly, the aim of the current study was to evaluate the possible neuroprotective effects of DAPA in guarding against scopolamine (SCO)-induced AD in rats *via* inhibiting RAS classical arm, and activating RAS protective arm, as well as PI3K/Akt pathway. DAPA administration suppressed ACE expression and reduced the levels of Ang II and AT_1_ in the hippocampi, while elevated the hippocampal levels of ACE2 and Ang 1–7 in addition to boosting MAS1 hippocampal expression. Of note, DAPA prominently augmented the level of PI3K. Also it enhanced the activity of protein kinase A. Furthermore, the hippocampal levels of caspase-3, cytochrome-c, interleukin-6 and nuclear factor kappa B were suppressed, while guanine nucleotide-binding protein alpha-3 level was elevated by DAPA administration. DAPA also attenuated the histopathological SCO-induced damage in rats and enhanced their recognition memory and learning in novel object recognition test and Y-maze spontaneous alternation test. Hence, the current results represent the first evidence for proving that DAPA-induced activation of the protective RAS arm suppressed the cognitive deficits in SCO-induced AD in rats.

## Introduction

Alzheimer’s disease (AD) is a neurodegenerative debilitating disorder characterized by multiple pathological manifestations, including amyloid-β (Aβ) plaques, neuroinflammation, and apoptosis (Zhang et al. 2024). Exploring potential therapeutic agents targeted at alleviating or delaying the onset and progression of AD is eagerly warranted. Scopolamine (SCO) is commonly used for inducing behavioral models for the purpose of investigating cognitive disorders as AD (San Tang 2019). The contribution of overactivated renin angiotensin system (RAS) to the pathological mechanism of AD is well established (Ababei et al. 2023a; Ribeiro et al. 2020).

Previous studies have reported that overactivation of the classical RAS axis; angiotensin converting enzyme/angiotensin II/ angiotensin II type 1 receptor (ACE/Ang II/AT_1_) leads to Aβ-induced apoptosis and neurodegeneration in AD (Ababei et al. 2023b; Muthuraman and Kaur 2016). In addition, AT_1_ receptor activation results in the induction of apoptotic cascade (Zheng et al. 2014) and the elevation of inflammatory proteins (Goel et al. 2018). Furthermore, AT_1_ diminishes phosphoinositide 3-kinase/ protein kinase B (PI3K/Akt) pathway which contributes to the regulation of neuronal survival and memory (Hers et al. 2011; Villapol and Saavedra 2015). The activation of PI3K/Akt pathway mitigates neurotoxicity by decreasing the level of apoptotic markers as cytochrome-c (CYC), and caspase-3 (Casp-3) (Shi et al. 2015). In addition, PI3K/Akt pathway plays a critical role in mitigating neuroinflammation that guards against AD (Cianciulli et al. 2020).

The protective RAS axis; angiotensin converting enzyme 2/angiotensin 1–7/ Mas receptor (ACE2/Ang 1–7/MAS1) opposes the harmful effects of classical axis overactivation in various disorders including AD (Awwad et al. 2019; Karnik et al. 2017). It has been reported that Ang 1–7 induced MAS1-dependent neuroprotective effects against cognitive deficits in rats (Deng et al. 2023). Activation of MAS1 by Ang 1–7 exhibits neuroprotection stimulated by PI3K/Akt pathway (Cheng et al. 2024). Also, the neurotoxic Aβ could be converted to neuroprotective one by ACE2 (Liu et al. 2014). Besides, impaired novel object recognition memory was documented in MAS1 knockout mice (Lazaroni et al. 2012). A positive correlation was previously documented between MAS1 activation and PI3K/Akt pathway (Zhou et al. 2022).

Thus, drugs that are capable of activating RAS protective arm can be repurposed as an intervention for AD. Dapagliflozin (DAPA), a sodium glucose cotransporter 2 (SGLT2) inhibitor, was primarily developed for treating diabetes and was reported later to activate ACE2 and generate Ang 1–7 (Awwad et al. 2025; Li et al. 2021). However, DAPA’s possible neuroprotective effects in guarding against SCO-induced AD have not been previously explored.

The current experimental study is the first work conducted to investigate the potential beneficial effects of DAPA-associated RAS protective arm activation on SCO-induced AD. The study evaluated behavioral, biochemical, and histopathological alterations associated with AD. Since RAS overactivation is involved in the mechanism of AD. Therefore; the possible neuroprotective effects of treatment with DAPA on altered components of RAS and neurological pathologic markers as a result of SCO-induced AD in rats was assessed. Furthermore, the relevance of RAS modulation to the reduction of inflammatory and apoptotic proteins for the purpose of mitigating AD was evaluated.

## Materials and methods

### Drugs and reagents

SCO and DAPA were obtained from Sigma Aldrich Company, USA. All other chemicals and reagents consumed in the study were of the highest purity.

### Animals

Aged male Sprague Dawley rats (8 months old; 350 ± 20 g) were obtained from Faculty of Pharmacy, Pharos University in Alexandria, Alexandria, Egypt and used to conduct the current study. Rats were acclimatized for a period of one week before performing any procedures with free access to rat chow and water. Housing of animals was in constant conditions of humidity (60 ± 10%), temperature (25 ± 2 °C), and alternating 12-h light/dark cycles within the experimental period. Any unnecessary maneuver or disturbance was avoided. Experimental work was carried out in compliance with the guidelines for the proper care and use of laboratory animals published by the US National Institutes of Health (NIH Publications No. 8023, revised 1978) and the recommendations approved by the Ethics Committee of Faculty of Pharmacy, Pharos University in Alexandria, Egypt. (PUA01202504273347).

### Experimental protocol

Animals were randomly allocated into four groups of eight rats each. Control group (I): rats served as normal controls and received daily intraperitoneal (I.P.) saline. SCO group (II): SCO-induced AD by I.P. daily administration of 1 mg/kg SCO for a period of four weeks (Bhuvanendran et al. 2018). DAPA group (III): rats were administered DAPA (1 mg/kg) orally (El-Sahar et al. 2020). SCO + DAPA group (IV): rats were administered I.P. SCO (1 mg/kg) and DAPA (1 mg/kg) orally one week after SCO administration and continued for a period of three weeks.

At the end of the experimental period, animals were anesthetized using desflurane and hippocampi were isolated. Part of hippocampi was fixed in formal-saline (10%) for histopathological examination using hematoxylin and eosin (H&E) stain, while the other part was immediately washed with saline and stored at -80 °C in order to detect the biochemical biomarkers.

### Behavioral studies

#### Novel object recognition (NOR) test

The performance of NOR test was for the purpose of evaluating novel object recognition ability. The apparatus used was a dark rectangular open wooden box with dimensions of 80 cm length, 50 cm width and 60 cm height. The used objects were 5 cm height cylinders and cubes. In between trials, any fecal debris was removed and arena, as well as, objects were cleansed using a 70% ethyl alcohol solution. The test included three consecutive phases: habituation phase, familiarization phase, and test phase. The habituation phase allowed for rats’ exploration of the apparatus 5 min without the presence of any objects. Then during familiarization phase, two familiar cylinders (identical objects) were placed diagonally in the wooden box and rat exploration of the familiar objects was allowed for 5 min before returning the rat into its cage. After 24 h retention interval, the test phase was conducted during which a familiar cylinder and a novel cube object were explored by the rat for 5 min. Exploration was considered positive when the rat sniffs or touches the object with its nose. To calculate the recognition index the following equation was applied:

Recognition index = exploring time of novel object / (exploring time of novel object + exploring time of familiar object) × 100% (Sumiyoshi et al. 2021).

#### Y-maze spontaneous alternation test

The Y-maze spontaneous alternation test was conducted according to the method applied by Wall and Messier (Wall and Messier 2002). The dimensions of the three plastic arms of the used Y-maze are; 45 cm length, 35 cm height and 10 cm width, tilted at 120◦ from each other. On the experimentation day, half an hour before performing the test, rats were habituated to the testing room where lights were avoided for raising the exploration behavior of rats on the Y-maze. The three arms of the Y-maze were labeled as A, B, and C. Each rat was introduced at the junction of the Y-maze and allowed to move freely in the maze for 8 min. Then spontaneous alternation was recorded. Rat’s entrance into each maze arm was counted as complete if hind paws of each rat were placed into the arm completely. Alternation was traced as a triplet set of subsequent entries into Y-maze three arms (i.e., ABC, BCA….). Finally, the calculation of the percentage of spontaneous alternation performance (SAP %) was performed by applying the following equation:

$$({\mathrm{Number}}\;{\mathrm{of}}\;{\mathrm{alternations)}}/({\mathrm{Total}}\;{\mathrm{number}}\;{\mathrm{of}}\;{\mathrm{arm}}\;{\mathrm{entries}} - 2) \times 100 $$

### Colorimetric hippocampal parameters

Acetylcholine (ACh) and choline were colorimetrically measured in hippocampi (ng/mg protein and pg/mg protein, respectively) by rat-specific kits according to instructions of the supplier.

### Enzyme-linked immunosorbent assay (ELISA) in hippocampi

Rat-specific ELISA kits were used according to the instructions provided by the manufacturer to detect biochemical parameters in hippocampal tissue homogenates after centrifugation at 5000 g for 20 min for the collection of supernatant in order to determine nuclear factor kappa B (NF-κB) level (Wuhan Fine Biotechnology Co. Ltd., Wuhan, Hubei, China) expressed as ng/mg protein, interleukin 6 (IL-6) level (Cloud-Clone Corp., USA) expressed as pg/mg protein, guanine nucleotide-binding protein alpha-3 (GNAI3) level (Biobool, Mongkok Kowloon, Hong Kong) expressed as pg/mg protein, Casp-3 level (Cusabio Life science, Wuhan, Hubei, China) expressed as ng/gm tissue, CYC level (Cusabio Life science, Wuhan, Hubei, China) expressed as ng/gm tissue, Ang 1–7 level (FineTest, Wuhan, Hubei, China) expressed as pg/mg protein, ACE2 level (FineTest, Wuhan, Hubei, China) expressed as ng/mg protein, AT_1_ level (FineTest, Wuhan, Hubei, China) expressed as pg/mg protein, Ang II level (FineTest, Wuhan, Hubei, China) expressed as pg/mg protein, protein kinase A (PKA) level (MyBioSource, SanDiego, CA, USA) expressed as ng/mg protein and phosphoinositide 3-kinase (PI3K) level (Cusabio Life science, Wuhan, Hubei, China) expressed as pg/mg protein.

### Western blotting analysis

Estimation of ACE and MAS1 protein expression in hippocampal tissues was carried out by using western blotting analysis. The application of ReadyPrepTM protein extraction kit was in accordance with the manufacturer’s instructions. Protein quantification was conducted by Bradford assay. Protein concentration; 20 µg of each specimen was added to an equal volume of 2x Lammli sample buffer that contained sodium dodecyl sulfate (4%), 2-mercaptoehtanol (10%), glycerol (20%), bromophenol blue (0.004%) and Tris HCl (0.125 mol/L). The pH was kept at 6.8. For the purpose of ensuring protein denaturation; boiling of the above mixture for 5 min was performed before being loaded on PAGE. Then to allow for the protein bands transference from gel to membrane, the blot was run. The membrane was blocked in tris-buffered saline with Tween 20 (TBST) buffer and bovine serum albumin (BSA) (3%) at room temperature. Primary antibodies were diluted by TBST. According to the manufacturer’s guidance, the chemiluminescent substrate (Clarity TM Western ECL substrate Bio-Rad Catalog # 170–5060) was added to the blot. Equal volumes of solution A and solution B; clarity western luminal/enhancer solution and peroxidase solution, respectively were added. Then signals were captured by a camera-based imager. Data expression was as arbitrary units after normalization for β-actin (housekeeping protein).

### Histopathological investigation

Hippocampal specimens of 4 μm thicknesses were stained with H&E, after being fixed in formal-saline (10%) and paraffin embedded, for examination under light microscope by blinded histopathologist.

### Statistical analysis

Version 7.0 GraphPad Prism software was used for results analysis. Expression of experimental values was as mean ± SE (*n* = 8). One-way analysis of variance test (one-way ANOVA) followed by Student-Newman-Keuls multiple comparison test were used for comparing between different groups. Results’ significance level was kept at *p* < 0.05.

## Results

### Effect of DAPA treatment on cognitive functions of SCO-administered rats in NOR and Y-maze behavioral tests

**Fig. 1:**
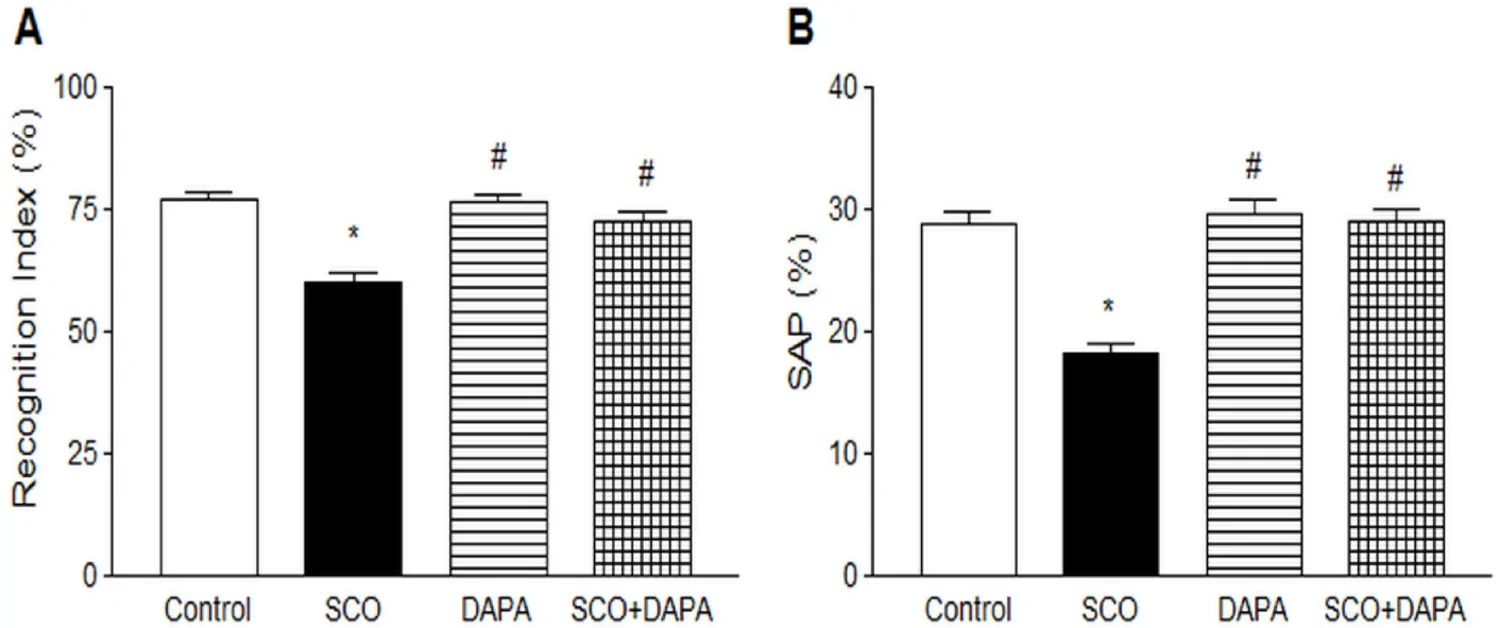
Effect of DAPA treatment on cognitive functions of SCO-administered rats in NOR and Y-maze behavioral tests. Effect of DAPA on SCO-induced alterations of the percentage of **A** recognition index in novel object recognition (NOR) test, and **B** spontaneous alteration performance (SAP) in Y-maze test. SCO, scopolamine; DAPA, dapagliflozin. Each bar represents the mean ± SE of 8 rats per group. Statistical analysis was performed using one-way ANOVA followed by Student-Newman-Keuls multiple comparison test; ^*^p < 0.05 versus control; ^#^*p* < 0.05 versus SCO

In NOR behavioral test, SCO-administered rats showed a significant lower recognition index than that of the control rats. SCO-administered rats treated with DAPA exhibited prominent increase in the recognition index as compared to SCO-administered rats. DAPA-only treated group showed similar results to that of the control group with no statistical significance between the two groups (Fig. 1A).

Y-maze test demonstrated that spontaneous alteration performance (SAP) in SCO-treated group was notably reduced as compared to control group. SCO+DAPA group demonstrated sharp increase in the rate of SAP with respect to SCO-treated rats. No statistical significant difference was shown between the control group and DAPA-only treated group (Fig. 1B).

### Effect of DAPA treatment on ACh and choline levels in hippocampal tissues of SCO-administered rats

**Fig. 2:**
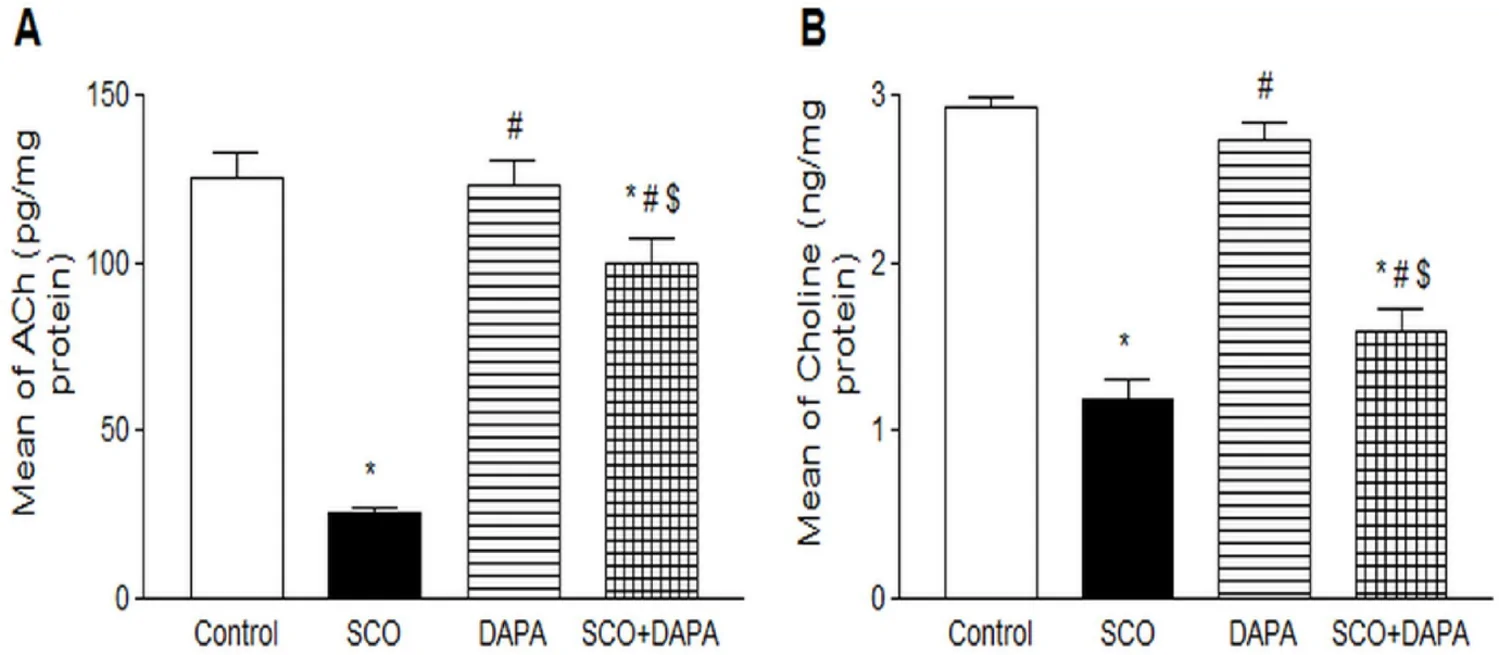
Effect of DAPA treatment on ACh and choline levels in hippocampal tissues of SCO-administered rats. Effect of DAPA on SCO-induced hippocampal alterations of **A** acetylcholine (ACh) level, and **B** choline level. SCO, scopolamine; DAPA, dapagliflozin. Each bar represents the mean ± SE of 8 rats per group. Statistical analysis was performed using one-way ANOVA followed by Student-Newman-Keuls multiple comparison test; ^*^p < 0.05 versus control; ^#^*p* < 0.05 versus SCO; ^$^*p* < 0.05 versus DAPA

ACh and choline levels were dramatically decreased in hippocampal tissues of SCO group with regards of control group. DAPA treatment of SCO-administered rats significantly elevated ACh and choline levels with respect to SCO-treated group. DAPA-only treated group showed similar results to that of control group with no statistical significance between both groups (Fig. 2A and B).

### Effect of DAPA treatment on inflammation in hippocampal tissues of SCO-administered rats

**Fig. 3:**
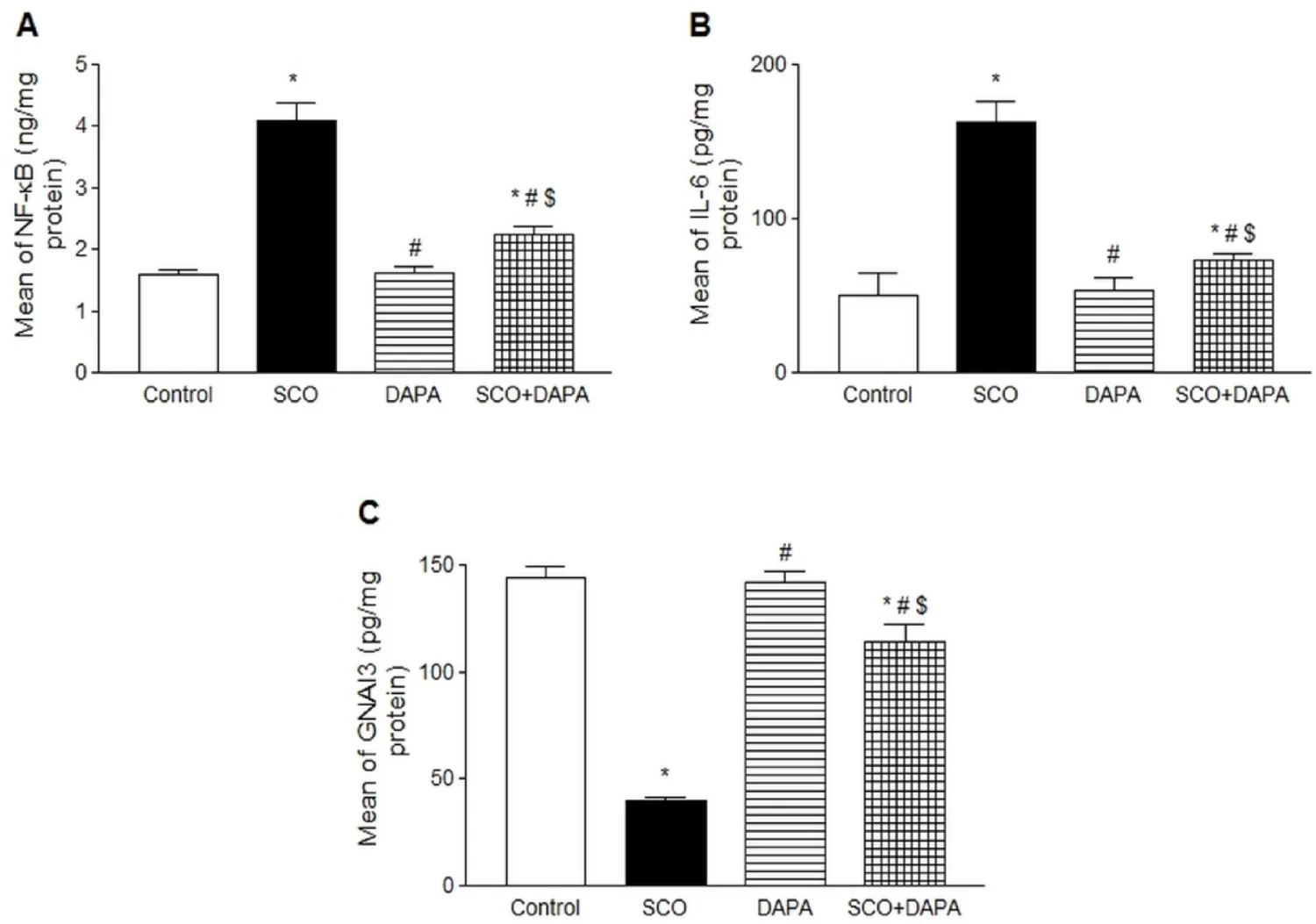
Effect of DAPA treatment on inflammation in hippocampal tissues of SCO-administered rats. Effect of DAPA on SCO-induced hippocampal alterations of **A** nuclear factor kappa B (NF-κB) level, **B** interleukin-6 (IL-6) and **C** guanine nucleotide-binding protein alpha-3 (GNAI3) level. SCO, scopolamine; DAPA, dapagliflozin. Each bar represents the mean ± SE of 8 rats per group. Statistical analysis was performed using one-way ANOVA followed by Student-Newman-Keuls multiple comparison test; ^*^p < 0.05 versus control; ^#^*p* < 0.05 versus SCO; ^$^*p* < 0.05 versus DAPA

SCO induced a significant rise in NF-κB and IL-6 proinflammatory proteins’ levels and a prominent decline in GNAI3 anti-inflammatory protein level with regards of the control group. DAPA treatment of SCO-administered rats profoundly decreased NF-κB and IL-6 levels, and notably elevated GNAI3 level as compared to SCO-administered group. DAPA-only treated group demonstrated comparable results to the control group (Fig. 3A–C).

### Effect of DAPA treatment on apoptosis in hippocampal tissues of SCO-administered rats

**Fig. 4:**
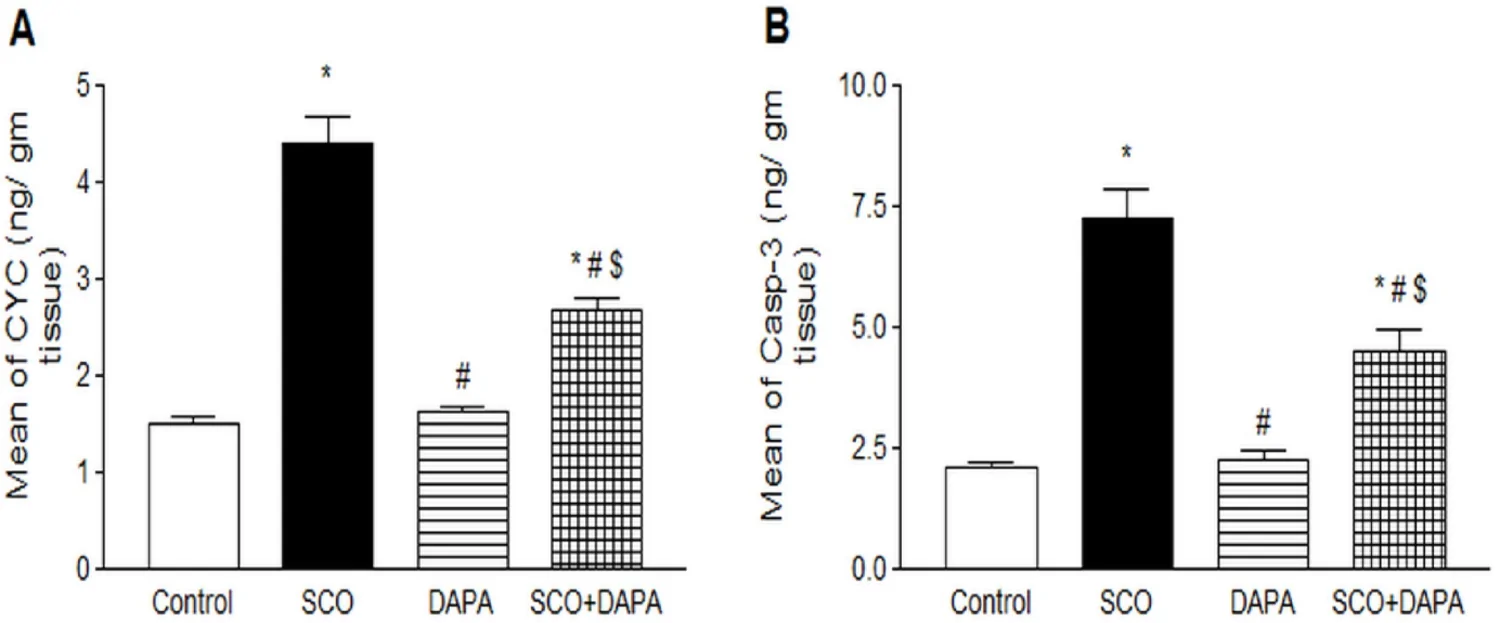
Effect of DAPA treatment on apoptosis in hippocampal tissues of SCO-administered rats. Effect of DAPA on SCO-induced hippocampal alterations of **A** cytochrome-c (CYC) level and **B** caspase-3 (Casp-3) level. SCO, scopolamine; DAPA, dapagliflozin. Each bar represents the mean ± SE of 8 rats per group. Statistical analysis was performed using one-way ANOVA followed by Student-Newman-Keuls multiple comparison test; ^*^p < 0.05 versus control; ^#^*p* < 0.05 versus SCO; ^$^*p* < 0.05 versus DAPA

The apoptotic markers CYC and Casp-3 were dramatically increased in SCO-administered rats with respect to control rats. DAPA use in SCO-administered rats significantly decreased CYC and Casp-3 levels as compared to SCO-administered group. Results of both DAPA-only treated group and control group were comparable (Fig. 4A and B).

### Effect of DAPA treatment on RAS components in hippocampal tissues of SCO-administered rats

**Fig. 5:**
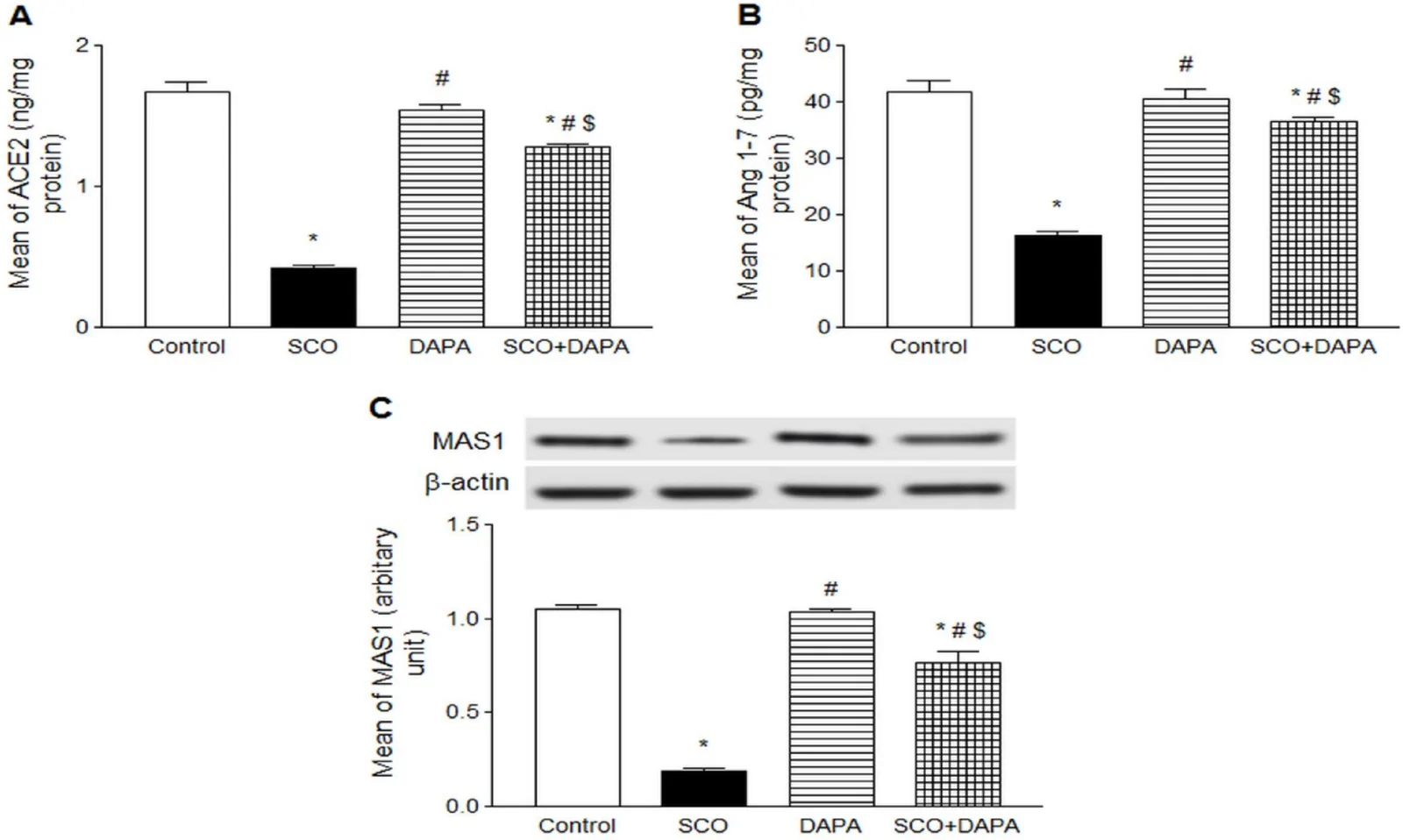
Effect of DAPA treatment on RAS protective arm components in hippocampal tissues of SCO-administered rats. Effect of DAPA on SCO-induced hippocampal alterations of **A** angiotensin converting enzyme 2 (ACE2) level, **B** angiotensin 1–7 (Ang 1–7) level, and **C** Mas receptor (MAS1) expression. SCO, scopolamine; DAPA, dapagliflozin. Each bar represents the mean ± SE of 8 rats per group. Statistical analysis was performed using one-way ANOVA followed by Student-Newman-Keuls multiple comparison test; ^*^p < 0.05 versus control; ^#^*p* < 0.05 versus SCO; ^$^*p* < 0.05 versus DAPA

**Fig. 6:**
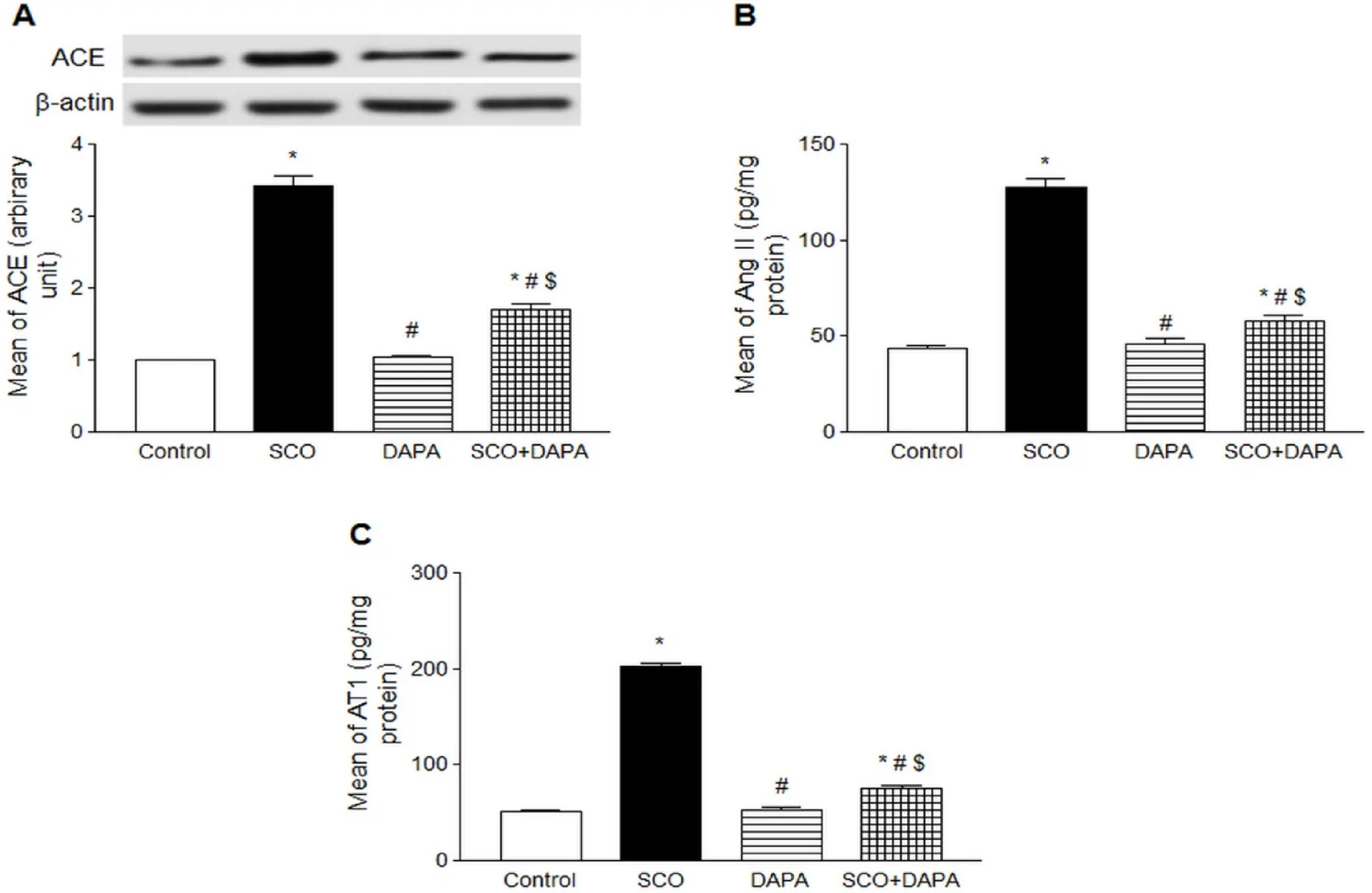
Effect of DAPA treatment on RAS classical arm components in hippocampal tissues of SCO-administered rats. Effect of DAPA on SCO-induced hippocampal alterations of **A** angiotensin converting enzyme (ACE) expression, **B** angiotensin II (Ang II) level, and **C** angiotensin II type 1 receptor (AT_1_) level. SCO, scopolamine; DAPA, dapagliflozin. Each bar represents the mean ± SE of 8 rats per group. Statistical analysis was performed using one-way ANOVA followed by Student-Newman-Keuls multiple comparison test; ^*^p < 0.05 versus control; ^#^*p* < 0.05 versus SCO; ^$^*p* < 0.05 versus DAPA

Hippocampal levels of ACE2 and Ang 1–7, as well as MAS1 expression were prominently reduced in SCO-treated group with respect to control group. DAPA treatment of SCO-administered rats resulted in a notable elevation in hippocampal levels of ACE2 and Ang 1–7, in addition to significant upregulation of MAS1 expression as compared to SCO-treated group. DAPA-only treated group showed similar results to that of control group with no statistical significance between both groups (Fig. 5A–C). Besides, SCO administration significantly elevated hippocampal levels of Ang II and AT_1_, as well as ACE expression with respect to control group. DAPA administration to SCO-administered rats showed significant reduction in elevated Ang II and AT_1_ levels, in addition to sharp downregulation of ACE expression in SCO-administered group. DAPA-only treated group showed similar results to that of control group with no statistical significance between the two groups (Fig. 6A–C).

### Effect of DAPA treatment on PKA and PI3K levels in hippocampal tissues of SCO-administered rats

**Fig. 7:**
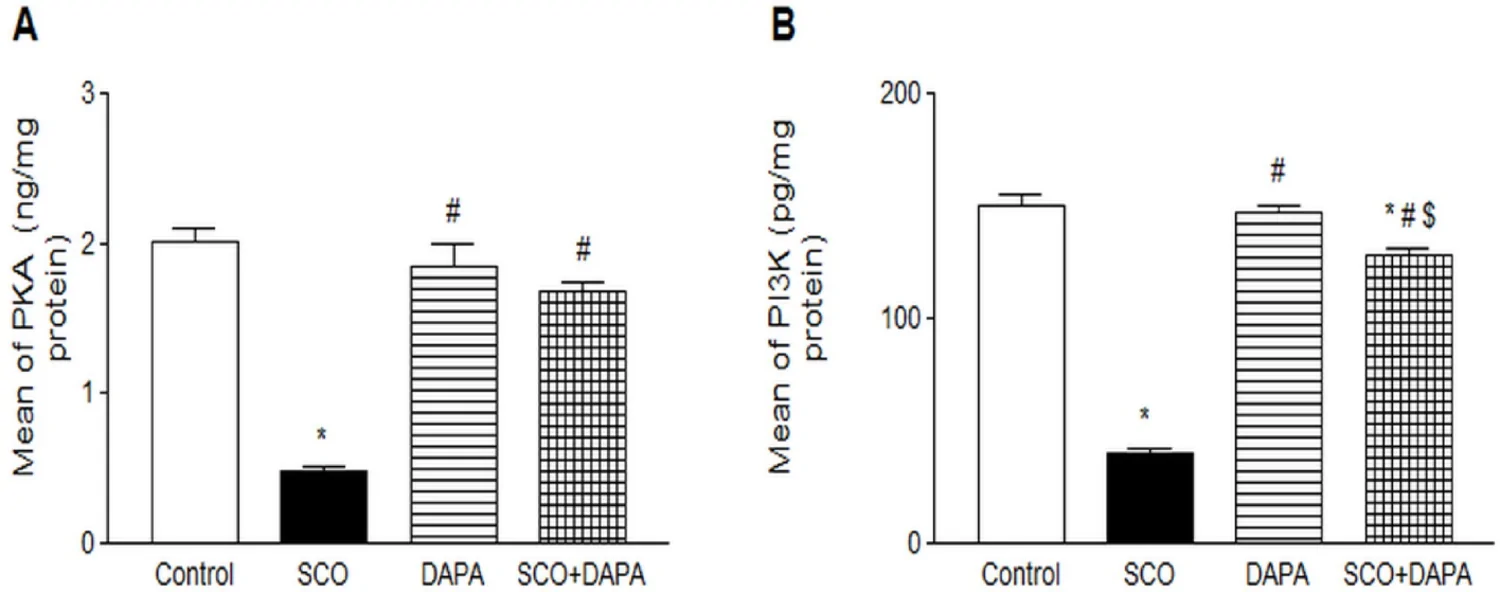
Effect of DAPA treatment on PKA and PI3K levels in hippocampal tissues of SCO-administered rats. Effect of DAPA on SCO-induced hippocampal alterations of **A** protein kinase A (PKA) level, and **B** phosphoinositide 3-kinase (PI3K) level. SCO, scopolamine; DAPA, dapagliflozin. Each bar represents the mean ± SE of 8 rats per group. Statistical analysis was performed using one-way ANOVA followed by Student-Newman-Keuls multiple comparison test; ^*^p < 0.05 versus control; ^#^*p* < 0.05 versus SCO; ^$^*p* < 0.05 versus DAPA

A sharp reduction in PKA level was exhibited in SCO group as compared to control group. DAPA treatment of SCO-administered rats prominently elevated PKA content as compared to SCO-administered rats. Moreover, SCO-administered rats demonstrated a dramatic reduction in PI3K level with regards of control rats. DAPA use in SCO-administered rats profoundly raised PI3K level with respect to SCO-administered rats. No statistical significant difference was shown between DAPA-only treated group and the control group (Fig. 7A and B).

### Effect of DAPA treatment on histopathological alterations in hippocampal tissues of SCO-administered rats

**Fig. 8:**
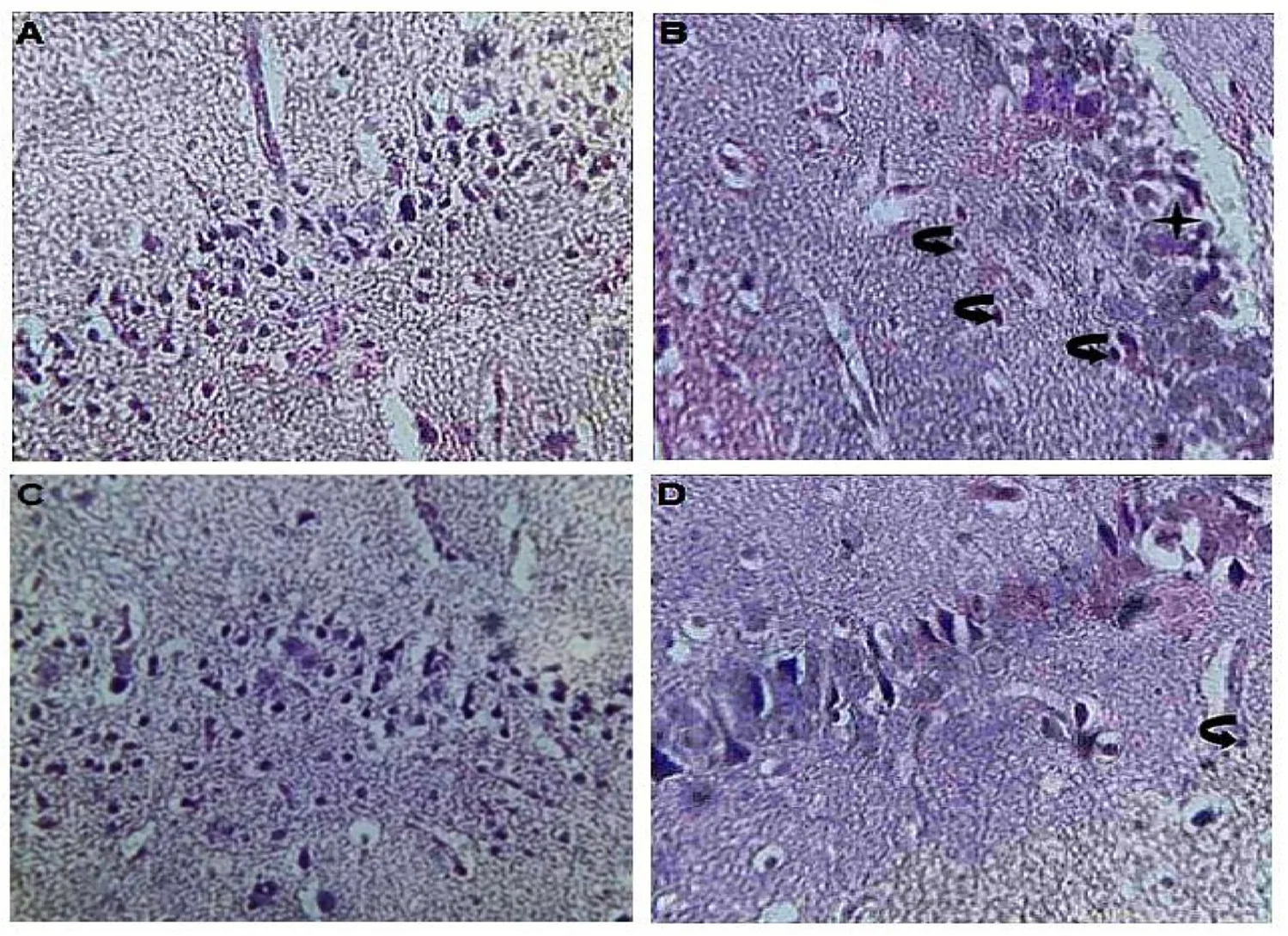
Effect of DAPA treatment on histopathological alterations in hippocampal tissues of SCO-administered rats. Photomicrographs of hippocampal rat tissue (H&E, x400). **A** Control rat demonstrating normal histological structure. **B** SCO-administered rats showing loss of pyramidal cells, vacuolation (star) and pyknotic neurons (curved arrow). **C** DAPA-only treated rat showing unchanged histological structure from the control rat. **D** SCO + DAPA-treated rat revealing mild pathological alterations of the pyramidal cells and few scattered pyknotic neurons

Normal fully integrated histological structure was shown in hippocampal sections of control rats (Fig. 8A). On contrary, loss of pyramidal cells, vacuolation (star) and pyknotic neurons (curved arrow) was revealed in SCO-administered rats (Fig. 8B). DAPA administration showed unchanged histological structure from the control rat (Fig. 8C). SCO + DAPA treatment resulted in mild pathological alterations of the pyramidal cells and less scattered pyknotic neurons as compared to SCO-administered rats (Fig. 8D) (H&E, × 400).

## Discussion

The present study documented that SCO use induced AD in rats *via* impairing the cholinergic pathway as elucidated by the decreased hippocampal levels of ACh and choline. Previous studies reported similar results (Ghallab et al. 2025; Toide 1989). While SCO-induced cholinergic receptor blockade represents a valuable means of inducing memory impairment, it does not fully mimic human AD. Key limitations of SCO-induced AD model include the inability of SCO to reproduce the progressive and multifactorial pathology of AD as unlike the natural progression of AD in humans, SCO-induced amnesia is primarily functional and lacks extensive widespread Aβ plaque aggregation (Jagielska et al. 2025), in addition to the absence of neurodegeneration as SCO induces reversible cognitive impairment (Wang et al. 2023), besides behavioral differences due to the failure of the model to capture motor activity changes in humans (Chintoh et al. 2003). However, based on the ability of SCO to induce cholinergic dysfunction and memory impairment, the current animal model could be used for the investigation of novel potential therapeutic agents for attenuating AD.

Behavioral tests of the present study demonstrated that SCO administration lead to a prominent decrease in the recognition index in NOR test, as well as a dramatic reduction of SAP% in Y-maze test indicating memory deficits as previously reported (Van Can et al. 2018; Wong-Guerra et al. 2017). Moreover, histopathological investigation illustrated loss of pyramidal cells, vacuolation and pyknotic neurons in SCO-administered rats. In parallel with the present findings, earlier studies have reported similar histopathological alterations in rats with AD (Patel et al. 2024; Samir et al. 2024).

Overactivation of the classical detrimental RAS axis is well-documented to underly the pathological mechanism of AD (de Miranda et al. 2024). It was previously proved that RAS inhibition exerts neuroprotective actions (Kalra et al. 2015; Rocha et al. 2018). The relationship between the cholinergic system and RAS was previously documented in AD. RAS inhibition was reported to reduce the blockade effects of cholinergic receptors (Wright and Harding 2010). An increased hippocampal expression of ACE, as well as Ang II and AT_1_ hippocampal levels were observed in SCO-administered rats. On contrary, SCO decreased hippocampal ACE2 and Ang 1–7 levels, in addition to MAS1 hippocampal expression as compared to control group.

Neuroinflammation and apoptosis are key processes in AD progression (Ghallab et al. 2025). SCO administration resulted in a prominent increase in the proinflammatory markers; NF-κB and IL-6, while dramatically decreased the antiinflammatory marker GNAI3. In addition, SCO lead to a significant increase in the apoptotic markers; CYC and Casp-3 that also evidenced the pathogenesis of AD. The observed elevation in proinflammatory and apoptotic markers aligns with several previous studies (Ghallab et al. 2025; Khodir et al. 2025). The elevated Casp-3 level might be attributed to the enhancement of CYC, as well as inflammatory cytokines (Snigdha et al. 2012). Also, Ang II is involved in mitochondrial dysfunction and CYC secretion that is counteracted by Ang 1–7 (Kim et al. 2012).

In addition, current findings showed that SCO administration dramatically decreased PI3K hippocampal levels that is matching with previous results that have demonstrated decreased levels of PI3K in postmortem AD cerebral specimens (Li et al. 2024; Wang et al. 2020). Moreover, SCO lead to a profound decrease in PKA level that has been proven to mediate AD (Bae et al. 2019; Wu et al. 2013).

A non-traditional strategy was investigated in the current study by using DAPA in SCO-administered rats for reviving the neuroprotective role of the beneficial arm of RAS in ameliorating AD. Traditional therapeutic agents that act by boosting cholinergic pathways in AD are used in the treatment of AD, however it was previously reported that cholinergic agents have limited efficacy in AD (Chen et al. 2022). As a result, novel alternatives need to be explored for discovering more efficacious therapies. Consequently, administration of DAPA to target RAS axes could be a promising therapeutic agent in ameliorating SCO-induced AD.

The current findings proved that DAPA treatment of SCO-administered rats guarded against SCO-induced AD as elucidated by the prominent elevation of choline and ACh hippocampal levels in SCO-administered rats. DAPA treatment of SCO-administered rats also lead to a profound increase in recognition index and SAP% with respect to SCO-administered group indicating the promising effects of DAPA in guarding against memory deficits. In accordance, previous experimental studies demonstrated improvement in behavioral dysfunction with DAPA treatment (Arab et al. 2023; El-Sahar et al. 2020; Gamal et al. 2025). Histological investigation showed a notable decrease in vacuolation, pyramidal cell loss and pyknotic neurons with DAPA use, suggesting its ameliorative effects against SCO-induced microscopic hippocampal abnormalities.

DAPA treatment of SCO-administered rats restored RAS balance as elucidated by the prominent increase in ACE2 and Ang 1–7 hippocampal levels, in addition to the increased hippocampal MAS1 expression. Concurrent reduction in hippocampal levels of Ang II and AT_1_, as well as ACE expression was also associated with DAPA use in SCO-administered rats. In support, previous studies have reported the augmentation of ACE2 and Ang 1–7 levels (Awwad et al. 2025; Li et al. 2021) and the reduction of ACE, Ang II and AT_1_ levels by DAPA administration (Ma et al. 2024; Urbanek et al. 2023).

Moreover, DAPA administration to SCO-administered rats dramatically reduced the inflammatory markers; NF-κB and IL-6, while significantly elevated the antiinflammatory marker GNAI3. Besides, DAPA treatment of SCO-administered rats lead to a profound decrease in the apoptotic markers; CYC and Casp-3. The neuroprotective impacts of DAPA against neurodegenerative diseases have been previously reported to be promoted by the ability of DAPA to reduce neuroinflammation and apoptosis, as well as to enhance synaptic plasticity, glucose regulation and the survival of neurons, owing to the presence of SGLT2 in brain that contributes to neuronal protection and fluid dynamics (Wiciński et al. 2020).

In parallel with the current results, MAS1-mediated mitigation of NF-κB has been previously reported to institute Ang 1-7-induced neuroprotection in stroke. Also, MAS1 upregulation and elevated ACE2 level in the hypothalamic paraventricular nucleus has attenuated IL-6 induced by AT_1_ (Sriramula et al. 2011). Activation of ACE2/Ang1-7/MAS1 axis has been documented to antagonize AT_1_-induced apoptosis by suppressing c-Jun N-terminal protein kinase phosphorylation which, consequently, inhibits Casp-3 (Uhal et al. 2011) and also MAS1 forms a heterodimer complex with AT_1_ that abrogates AT_1_ signaling (Pernomian et al. 2015).

The positive effects of DAPA on SCO-induced AD could be related to its capability of restoring the normal balance of RAS; this was reported, for the first time, in the current study. Although it has been previously documented that DAPA potentiates the protective RAS arm (Awwad et al. 2025), no earlier studies have reported its neuroprotective role against SCO-induced AD.

Results of the present work revealed that DAPA administration to SCO-administered rats sharply increased PI3K hippocampal level that could be associated with the increased Ang 1–7 level and MAS1 upregulation. It was previously reported that activation of MAS1 by Ang 1–7 activates PI3K/AKT pathway (Cheng et al. 2024) that, in turn, stimulates nitric oxide synthase. This pathway has been previously documented to promote neuronal survival *via* increasing the level of nitric oxide that enhances cerebral blood flow (Bennion et al. 2015; Santos et al. 2017).

Since, PKA decreased level contributes to the pathogenesis of AD. Hence, PKA plays a protective role against AD (Dagda and Tania 2015). The current findings documented that DAPA treatment of SCO-administered rats resulted in a prominent rise in PKA level that might be a result of Ang 1–7 potentiation *via* stimulating MAS1 (Karnik et al. 2017). PKA, subsequently, mediates structural and functional alleviation of synaptic proteins, and helps in the stimulation of memory preservation (Cui et al. 2019).

The retrieved results revealed, exclusively, that DAPA treatment exerted a neuroprotective effect against SCO-induced AD not only by enhancing the cholinergic pathway, but also by acting on RAS to restore the normal balance elucidating the promising effects of repurposing SGLT2 inhibitors, as well as RAS modulating drugs for ameliorating AD.

## Conclusion

In conclusion, the present work highlighted the detrimental effects of SCO on memory deficit in animals. This was evidenced by the dramatic decrease in choline and ACh hippocampal levels, in addition to the sharp reduction of recognition index and SAP percentage upon SCO administration. RAS imbalance, neuroinflammation and apoptosis are involved in the underlying pathophysiological mechanism of AD. The current study provided the first mechanistic evidence for the potential neuroprotective effects of DAPA in mitigating SCO-induced AD partially *via* shifting RAS balance toward the antiinflammatory, antiapoptotic and neuroprotective axis. Hence, novel therapeutic strategies targeting ACE2/Ang1-7/MAS1 activation in AD are promising modalities for guarding against AD.

## Data Availability

Data that support the findings of this study are available on request.
